# Genetic Mapping and Identification of the Candidate Gene for White Seed Coat in *Cucurbita maxima*

**DOI:** 10.3390/ijms22062972

**Published:** 2021-03-15

**Authors:** Yuzi Shi, Meng Zhang, Qin Shu, Wei Ma, Tingzhen Sun, Chenggang Xiang, Changlin Wang, Ying Duan

**Affiliations:** Key Laboratory of Biology and Genetic Improvement of Horticultural Crops of Ministry of Agriculture and Rural Affairs, Institute of Vegetables and Flowers, Chinese Academy of Agricultural Sciences, Beijing 100081, China; yanyeshi0411@gmail.com (Y.S.); mengzhang604@gmail.com (M.Z.); Shuq2719@gmail.com (Q.S.); wma20801@gmail.com (W.M.); suntingzhen91@gmail.com (T.S.); Chenggangxiang0527@outlook.com (C.X.)

**Keywords:** seed coat color, map-based cloning, phenylpropanoid/flavonoid metabolism pathway, *Cucurbita maxima*

## Abstract

Seed coat color is an important agronomic trait of edible seed pumpkin in *Cucurbita maxima*. In this study, the development pattern of seed coat was detected in yellow and white seed coat accessions Wuminglv and Agol. Genetic analysis suggested that a single recessive gene *white seed coat* (*wsc*) is involved in seed coat color regulation in *Cucurbita maxima*. An F_2_ segregating population including 2798 plants was used for fine mapping and a candidate region containing nine genes was identified. Analysis of 54 inbred accessions revealed four main Insertion/Deletion sites in the promoter of *CmaCh15G005270* encoding an MYB transcription factor were co-segregated with the phenotype of seed coat color. RNA-seq analysis and qRT-PCR revealed that some genes involved in phenylpropanoid/flavonoid metabolism pathway displayed remarkable distinction in Wuminglv and Agol during the seed coat development. The flanking InDel marker S1548 was developed to predict the seed coat color in the MAS breeding with an accuracy of 100%. The results may provide valuable information for further studies in seed coat color formation and structure development in Cucurbitaceae crops and help the molecular breeding of *Cucurbita maxima*.

## 1. Introduction

*Cucurbita maxima* is a worldwide economic crop in *Cucurbita* species used mainly for the consumption of the flesh and edible seed [1,2]. The kernel of edible seed pumpkin in *Cucurbita maxima* is rich in unsaturated fatty acids, amino acid, natural antioxidants, and other nutrients [3,4]. The predominant fatty acids in pumpkin seed are linoleic, oleic, palmitic, and stearic acid with high oxidative stability that would be suitable for food and industrial applications [5,6].

Seed coat color is an important agronomic trait in seed oil crops such as *Brassica napus*, *Glycine max*, and *Medicago truncatula*. The seed coat color and thickness are related to the efficiency of seed processing [7]. Compare with dark-colored seed coat, light-colored seed is a desirable trait with great potential for improving seed quality [8,9]. In the edible seed genetic breeding, the varieties displaying white and thin seed coat are preferred for removing the seed coat conveniently and display a high kernel yield [10].

The seed coat displayed a wide range of colors such as brown, yellow, ivory, and white in *Cucurbita* species [10,11]. Seed coat color has been extensively studied in Arabidopsis and several oil crops such as *Brassica napus*, *Glycine max*, and some species in Cucurbitaceae [12]. In Arabidopsis, the seed coat arises from the integument of the maternal ovule and mainly develops into five layers. The integument layers include endothelium and two adjacent layers. The outer integument layers include subepidermal layers and epidermal cell layers [13,14]. A secondary thickened cell wall is formed in subepidermal cell layers and is deposited around the epidermis layers. Seed coat structure observed in *Cucurbita* species are referred as chlorenchyma, parenchyma, sclerenchyma, hypodermis, and epidermis [15,16]. The normal seed coat hypodermis, sclerenchyma, and parenchyma tissues are lignified gradually in the development of seed coat [15]. After ripening, the outer side of seed coat will dehydrate and form a high lignified shell to protect the seeds from stress of external environmental factors.

Flavonoids are secondary metabolites in phenylpropanoid pathway. Flavonols, anthocyanins, and proanthocyanidins are the three main flavonoids [17]. Proanthocyanidins are also known as concentrated tannic acid, and the seed coat is brown when the proanthocyanidins are oxidized [13]. Flavonoid metabolisms (predominantly tannins) are produced in endothelium and deposited to form brown seed coat after oxidization in mature seeds [12,14,18,19]. By analyzing the components of different color seed coats in Arabidopsis, *Brassica rapus*, and other plants, the key enzyme regulation steps from phenylpropanes to proanthocyanidins have been determined clearly [20]. The initial substrates for the synthesis of flavonoids are derived from coumaroyl-CoA of phenylpropane metabolic pathways. Serial components in seed coat pigments synthesis have been identified in flavonoid metabolism pathway including Chalcone synthase (CHS), Chalcone isomerase (CHI), Flavonoid 3′-hydroxylase (FLS), Dihydroflavanol-4-reductase (DFR), Anthocyanin synthase (ANS), and Leucoanthocyanins reductase (LAR) [17,21]. Arabidopsis *transparent testa* (*tt*) genes encoding proteins involved in flavonoid metabolism display important roles in seed coat color formation and seed structures [22,23,24]. *tt* mutants display light seed coat color, defect in seed coat permeability and lower dormancy [25]. *TT* genes are regulated by MYB-bHLH-WD40 (MBW) protein complex. MBW is composed of R2R3-MYB, bHLH transcription factor, and WD40-repeat family members [12,26,27]. Among them, R2R3-MYB family transcription factors play a critical role in the production of anthocyanins or proanthocyanidins [28].

In Cucurbitaceae species, compared to melon and cucumber, watermelon and pumpkin/squash display rich diversity on seed traits including seed size, seed weight, and seed colors, which seem to depend on the intended uses and under a significant artificial selection in modern breeding [10]. At present, genetic analysis and QTL localization of seed coat color has been studies in Cucurbitaceae crops such as melon and watermelon. In melon, the white seed coat is a quality trait, and it is dominant to yellow and brown [29]. In watermelon, the seed coat color was determined by four loci (*r*, *w*, *t* for red, white, and tan, respectively; *d* for producing a black and dotted seed coat) which have been identified on chromosome 3, 5, 6, 8 [7,30,31]. The light cream color was recessive to the brown or red seed coat color, and black color was dominant over brown light cream and red color of seed coat separately or independently [32]. Several QTLs associated with black and light yellow seed coat were located on chromosome 1, 3, and 8 [30]. The candidate genes for qsc-c3-1 associated with black seed coat color was narrowed to a 70.2 kb region and a gene encoding Polyphenol oxidase (PPO) was identified to associate with black seed coat [30]. However, the inheritance of seed coat color was not very clear in *Cucurbita maxima*.

Recently, great efforts have been made in genetic linkage map construction and genome information in *Cucurbita* species. Molecular markers including random amplified polymorphic DNA (RAPD), amplified fragment length polymorphism (AFLP), simple sequence repeat (SSR), and single-nucleotide polymorphism (SNP) have been developed to construct genetic linkage map in *Cucurbita* [33,34,35]. Improvements of whole genome sequences and high-density genetic maps have promoted the precision of genomic data in *Cucurbita* and support a better foundation for the research and application of candidate genes in *Cucurbita* species [36,37,38,39,40,41]. With effective utilization of molecular markers and genetic map resources, QTLs for many agronomic traits and candidate genes have been identified and applicated accordingly in breeding selection [42,43,44,45,46]. Some QTLs of seed trait such as seed size, seed width, and seed yields have been identified in *Cucurbita maxima* and candidate genes are precited [10,47]. As one of the most important traits in *Cucurbita maxima*, studies about genetic and molecular mechanism of seed coat color have not been elucidated clearly.

In this study, two inbred accessions displaying white and yellow seed coat color were used to identify the pigment components of seed coat color formation in *Cucurbita maxima*. The inheritance of seed coat color was analyzed and a candidate gene encoding MYB transcription factor was predicted by map-based cloning. The molecular marker associated with the phenotype of seed coat color has been developed for MAS. The present results may highlight the understanding of the genetic basis for seed coat color formation and help further functional research in *Cucurbita maxima*.

## 2. Results

### 2.1. The Pigment Deposition of Seed Coat in Cucurbita maxima

**Figure 1:**
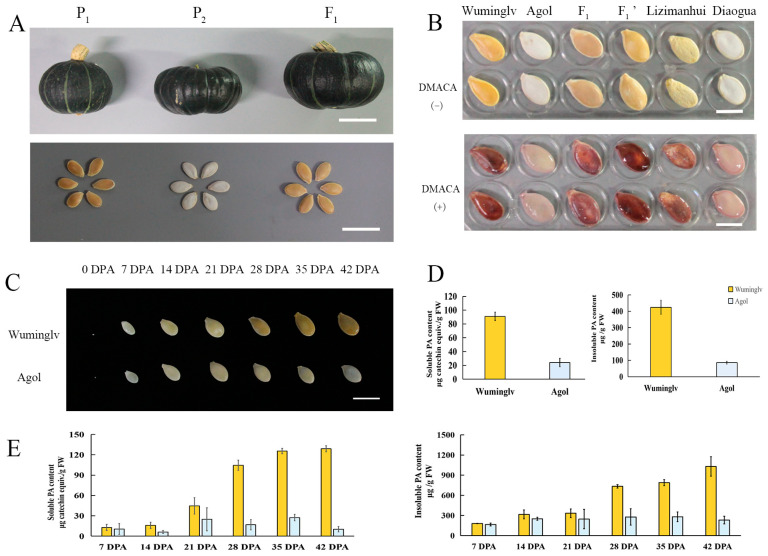
The phenotype of seed coat color and pigment accumulation in *Cucurbita maxima.* (**A**) Fruit and seed coat color in Wuminglv and Agol. The upper bar = 5 cm, the lower bar = 2 cm. (**B**) DMACA staining in Wuminglv (P_1_), Agol (P_2_), F_1_, F_1_′, Lizimanhui (P_3_), and Diaogua (P_4_). Bar = 2 cm. (**C**) Seed coat color formation during the seed development at different stage after pollination. Dynamic changes of seed coat color at 0, 7, 14, 21, 28, 35, 42 DPA (days post anthesis). Bar = 2 cm. (**D**) Soluble and insoluble PA determination of mature dry seeds in Wuminglv and Agol. (**E**) Soluble and insoluble PA determination of flesh seeds at 7, 14, 21, 28, 35, 42 DPA in Wuminglv and Agol.

Seed coat color is a very important agronomic trait of edible seed pumpkin in *Cucurbita maxima*. In this study, the seed coat color of 54 inbred accessions were observed and categorized into white or yellow. Twenty-three inbreds displaying white and 31 inbreds displaying yellow seed coat were obtained. Wuminglv (yellow seed coat, P_1_), Agol (white seed coat, P_2_), Lizimanhui (yellow seed coat, P_3_), and Diaogua (white seed coat, P_4_) were selected as parental lines and crossed to produced genetic populations (Figure 1A,B, Supplement Figure S1). At 0 DPA (Day Post Anthesis) of Wuminglv and Agol, the seed coat colors were both transparent. At 21~28 DPA, the seed coat color of Wuminglv was transferred into yellow, while Agol still remained white, suggesting 21~28 DPA was the important period of seed coat color formation in *Cucurbita maxima* (Figure 1C). L*, a*, and b* values and the comprehensive chromaticity E were obtained by chromometer to eliminate the deviation of vision subjective understanding of color. The L* color value in white seed coat ranged from 79.6 to 92.79, while that in yellow seed coat ranged from 60.39 to 72.29. The color value in BC_1_, BC_2_, and two F_2_ segregating populations displayed an expected arrangement according to the distribution of separation ratio (Table 1).

The color of petal and of flowers displayed yellow and the difference was not very obvious in Wuminglv and Agol (Supplement Figure S2A), followed by a slight difference in flesh color after fruit maturation (42 DPA) (Supplement Figure S2B,C).

DMACA (4-(Dimethylamino) cinnamaldehyde) is used as an indicator of procyanidins and flavan-3-ol [14,24,48]. To determine the composition of color pigment of seed coat qualitatively, DMACA staining was conducted in Wuminglv, Agol, Diaogua, Lizimanhui, and 76 inbred accessions including 29 white and 47 yellow seed coat accessions. The accessions displaying yellow seed coat were stained dark red and the accessions displaying white seed coat were stained rarely, which was closely related to the seed coat color (Figure 1B, Supplement Figure S3). Dynamic analysis of soluble and insoluble PA in different stages of seed development indicated that the content of procyanidins in the seed coat was significantly different at 21 DPA in Wuminglv and Agol, which is in accordance with the observation of seed coat color (Figure 1D,E).

### 2.2. The Histochemical Analysis of Seed Coat

**Figure 2:**
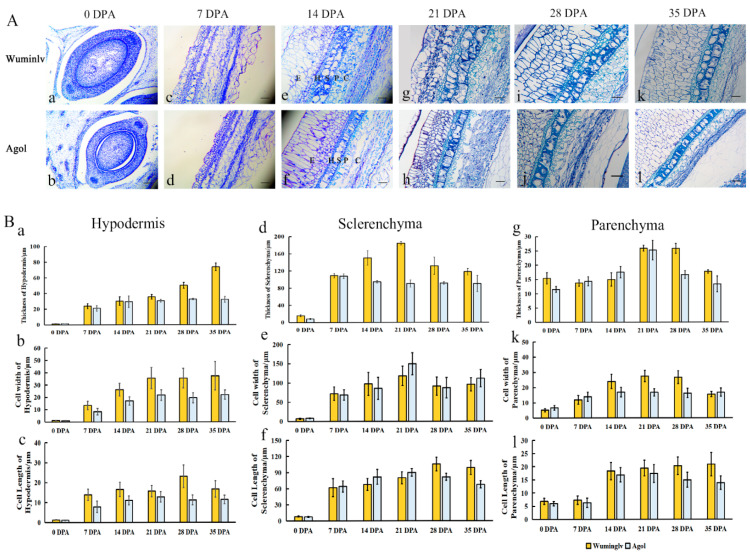
Histochemical analysis of the seed coat at different stage of seed development. (**A**) Morphological structures of seed coat. Dynamic changes of seed coat in Wuminglv and Agol at 0 (a, b), 7 (c, d), 14 (e, f), 21(g, h), 28 (i, j), 35 (k, l) DPA. Different characters represented cell layers as Epidermis (E), Hypodermis (H), Sclerenchyma (S), Parenchyma (P), and Chlorenchyma (C). Bar = 100 μm. (**B**) The statistics analysis of cell layers of hypodermis (a, b, c), sclerenchyma (d, e, f), and parenchyma (g, h, l).

To determine the pigment accumulation and differentiation layers of seed coat, anatomic structure was observed at 0, 7, 14, 21, 28, 35 DPA seed coats in Wuminglv and Agol (Figure 2A). At 14 DPA, there were five layers of seed coat: Epidermis (E), Hypodermis (H), Sclerenchyma (S), Parenchyma (P), and Chlorenchyma (C) (Figure 2(Ae,Af)). At 14~21 DPA, the cell differentiation in S layer displayed distinguish in Wuminglv and Agol (Figure 2(Ae–Ah)). The statistical analysis of cell width, cell length, and layers thickness in H, S, and P layers were calculated (Figure 2B). The thickness of seed coat was not obvious at the early stage of development (0~14 DPA), but the structure of H and S layers displayed significant differentiation where the pigment deposition at 21~28 DPA. There were two S-cell layers in Wuminglv but only one S-cell layer in Agol. Toluidine blue staining revealed that the polymeric phenolic compounds in Wuminglv were stained more intensively than that in Agol at 14~21 DPA, followed a dramatic difference at 28~35 DPA (Figure 2(Ai–Al)). As lignin was one of the important polyphenolic compounds in the secondary cell wall formation, the content of seed coat lignin was determined by phloroglucinol staining and thiogylcolic acid method (Supplement Figure S4A,B). The results indicated that more lignin was accumulated at the mature seed stage in Wuminglv, in accordance with a secondary cell wall thickened in S layers, compared to that in Agol.

### 2.3. Genetic Analysis of Seed Coat Color

Two genetic populations were used to determine the inheritance of seed coat color. For Wuminglv (P_1_) and Agol (P_2_), a six-generation population including P_1_, P_2_, F_1_, BC_1_, and BC_2_ was constructed and the seed coat color was determined by visual method and chromometer. As visual observation, all F_1_ plants exhibited the same seed coat color as Wuminglv. In 377 individuals of an F_2_ segregating population, 282 plants displayed yellow and 95 plants displayed white seed coat. The segregation pattern fit a Mendelian ratio of 3:1 (χ2 = 0.002, *p* < 0.05). In the 121 individuals of BC_2_ population, 65 plants displayed yellow seed coat and 56 plants displayed white seed coat. Statistical analysis showed that the segregating pattern of BC_1_P_2_ fit 1:1 separation ratio (χ2 = 0.17, *p* < 0.05) (Table 2). The similar results were displayed in the L*, a*, and b* determination (Supplement Table S1). For Lizimanhui (P_3_) and Diaogua (P_4_), similar inheritance was displayed in F_2_ segregating population (Supplement Table S2). The results indicated that the phenotype of seed coat color was controlled by a recessive single gene and was named as *white seed coat* (*wsc*).

### 2.4. Map-Based Cloning of White Seed Coat

**Figure 3:**
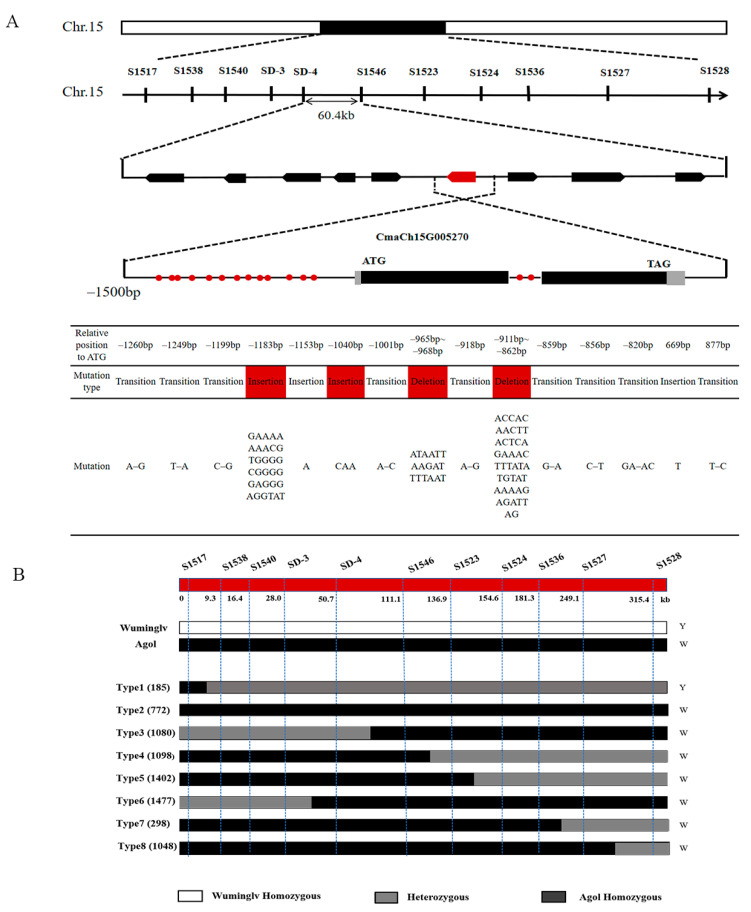
Map-based cloning of *WSC.* (**A**) Map-based cloning of *WSC* to 60.4 kb on Chromosome 15 in *Cucurbita maxima*. Sequence analysis in the promoter region of *CmaCh15G005270*. Red box represented two insertions and two deletions in −1300~−800 bp of the promoter region in Wuminglv. **(B**) Recombinants analysis for map-based cloning of *WSC*. White and black columns represented homozygous fragments identity with Wuminglv background (white columns) and Agol background (black columns), respectively. Gray columns represented heterozygous fragments. The numbers in brackets indicated the accession number of the recombined plants derived from F_2_ segregating population.

The whole genome resequencing (20× coverage) of Wuminglv and Agol were performed on Illuminar Hiseq platform and 324 InDel markers were designed evenly distributed every 1 Mb on each chromosome. One hundred and twenty-five InDel markers displayed clear and stable bands and polymorphisms consistent with two parents and F_1_ were selected to analyze an F_2_ segregating population of 2798 individuals. *WSC* was located at Chromosome 15 in primary mapping. Using two flanking markers S1517 and S1528 at a distance of 315.4 kb, 34 recombinants were identified and the phenotype of each recombinant was confirmed by F_2:3_ segregating population (Figure 3A). Forty-two novel InDel markers and 11 dCAPS markers were designed in this region based on the whole genome sequencing (Supplement Table S3). Seventeen InDel markers and nine dCAPS markers displayed polymorphisms among Wuminglv and Agol. The candidate region was ultimately narrowed in 60.4 kb interval between dCAPS marker SD-4 and InDel marker S1546 (Figure 3B).

### 2.5. Candidate Gene and Expression Analysis

There were nine genes in the candidate region (Table 3) including vesicle-associated membrane protein, MYB transcription factor, pentatricopeptide repeat-containing protein, unknown gene, and some other genes. The function of some genes has been studied in Arabidopsis and other crops, such as *CmaCh15G005220*/*vesicle-associated membrane protein 727* and *CmaCh15G005270*/*AtDIV2* [49,50]. From the gene annotations, few information of genes was related to seed coat color formation or pigment synthesis.

As *CmaCh15G005270*, *CmaCh15G005280*, *CmaCh15G005290*, and *CmaCh15G005300* were considered as the candidate gene, the full length of each gene including promoter, 5′-UTR and 3′-UTR was sequenced in Wuminglv and Agol. In *CmaCh15G005270*, there were two insertions, two deletions, and nine mutations presented in Wuminglv vs. Agol in a region of –1300~–800 bp from the initiation site. Meanwhile, one T insertion and one T–C transition were found in the first intron of the gene (Figure 3A).

To confirm whether these variant sequences were linked to seed coat color, the –1300~–800 bp region of the promoter were sequenced in 54 accessions with different genetic background. Three variant types of promoter sequence were found in 23 white accessions and named Type-Agol (21 accessions), Type 1 (one accession, No. 308), and Type 2 (one accession, No. 264) (Supplement Figure S5).

**Figure 4:**
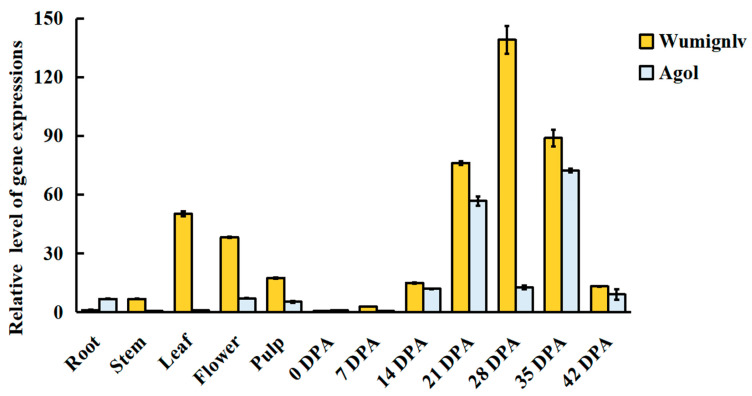
Transcript expression of *CmaCh15G005270* in root, stem, leaf, flower, pulp, and different development stage of seed coat in Wuminglv and Agol. *CmaCh15G005270* was highly expressed in leaf, flowers, pulp, and seed coat in Wuminglv.

Gene annotation indicated that *CmaCh15G005270* was predicted to encode an R2-MYB transcription factor homolog with *DIVARICATA2* (*ATDIV2*, *At5g04760*) and involved in the salt and ABA signaling in Arabidopsis. *DIV1* displayed a critical role for determining ventral identities in *Antirrhinum* corolla [51,52]. qRT-PCR showed that the candidate gene *CmaCh15G005270* was highly expressed in flowers and flesh, with the highest expression level in the seed coat at 28 DPA, and then gradually decreased (Figure 4). The results showed *CmaCh15G005270* may be the candidate gene in regulating the seed coat color in *Cucurbita maxima*.

### 2.6. RNA-Seq Analysis

To further determine the metabolic network and downstream regulatory genes involved in seed coat color formation and seed coat development, RNA-seq was conducted in 0, 7, 14, 21, 28, 35, 42 DPA seed coats in Wuminglv and Agol. Three biological duplicates were conducted at each time course and 312.27 G data was obtained with a Q30 value ranging from 90.57% to 94.27% (Supplement Table S4).

**Figure 5:**
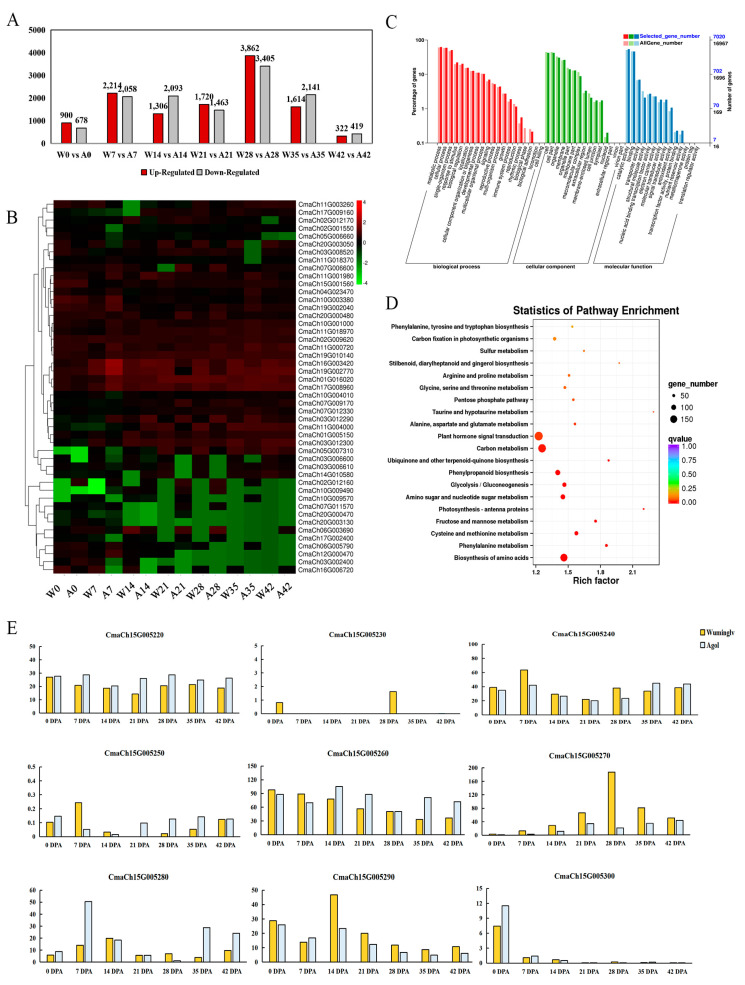
RNA-seq data analysis of phenylpropanoid/flavonoid metabolism pathway. (**A**) The amount of DEGs in different stages of seed coat development. (**B**) Heatmap of DEGs in phenylpropanoid/flavonoid metabolism pathway at 0~42 DPA seed coat. (**C**) GO enrichment analysis. (**D**) KEGG enrichment analysis. (**E**) Transcript expression levels in nine candidate genes in different stages of seed coat development in Wuminglv and Agol.

At all the development periods, the number of up-regulated DEGs in Wuminglv was ranged from 322 to 3862, and the number of down-regulated DEGs was from 419 to 3405, compared to Agol. The amount of DEGs was most remarkable at 28 DPA, in accordance with the period of seed coat color formation (Figure 5A,B). GO enrichment analysis showed that biological processes such as metabolic process, physiological process, antioxidant activity, and nuclear acid binding transcription factor activity were significantly enriched during the development of seed coat (Figure 5C). KEGG enrichment analysis indicated phenylalanine metabolism and phenylpropanoid biosynthesis were clustered significantly (Figure 5D). The difference of seed coat color in Wuminglv and Agol was closely related to the de novo biosynthesis pathway of phenylpropanoid.

**Figure 6:**
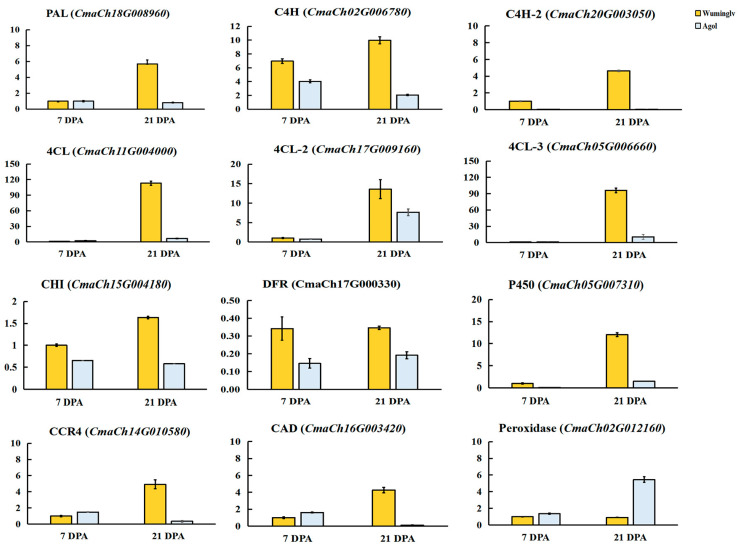
Expression patterns of twelve genes involved in phenylpropanoid/flavonoid metabolism pathway in Wuminglv and Agol seed coat.

The expression of some genes involved in flavonoid metabolism was confirmed by qRT-PCR in Wuminglv and Agol. The result of expression was consistent with that of RNA-seq data, indicating the reliability of RNA-seq (Figure 6). *PAL*, *C4H*, *4CL*, *CHI*, and *DFR* involved in phenylpropanoid/flavonoid were displayed remarkable relative expression differences in Wuminglv and Agol. RNA-seq data revealed that the transcript level of *CmaCh15G005270* increased gradually and arrived at a peak at 28 DPA. Meanwhile, the expression of this gene in Wuminglv was higher than that in Agol at all periods in seed coat development (Figure 5E). The results of RNA-seq illustrated that the phenylpropanoid/flavonoid metabolism pathway was affected in Wuminglv and Agol.

### 2.7. Validation of Molecular Marker

Fifty-four accessions (31 accessions of yellow seed coat and 23 accessions of white seed coat) and 222 F_2_ segregating population and RILs plants (107 accessions of yellow seed coat and 115 accessions of white seed coat) derived from P_3_ and P_4_ were used to validate the accuracy of the closest flanking InDel marker S1548 (Supplement Table S5). In these accessions, all of the accessions displaying yellow and white seed coat matched Wuminglv and Agol, suggesting that the accuracy of S1548 marker was 100% and exhibited validation in the molecular assistance breeding program in edible seed pumpkin in *Cucurbita maxima*.

## 3. Discussion

Seed coat color is one of the most important traits in oil seed and edible seed crops such as rape, soybean, and watermelon. In some crops, there was correlation between the seed quality, flesh appearance, and seed coat color [9]. In *Glycine max*, the distribution and variation of metabolites in seeds displayed a linkage with seed coat color [12,53,54]. The isoflavones (including malonylglucosides, glucosides, aglycone, and acetylglucosides) were gradually decreased in green, yellow, black, and brown soybeans. Only anthocyanins (cyanidin-3-O-glucoside, delphinidine-3-O-glucoside, and petunidin-3-O-glucoside) were examined in black seed coat cultivars [53]. The isoflavones were associated with seed maturity while fatty acids, glucose, fructose, and sucrose were less linked with seed maturity [55]. In this study, the insoluble PA and soluble PA in yellow seed coat accession Wuminglv was higher than that in white seed coat accession Agol, indicating that flavonoid metabolisms contributed to the seed coat color formation. The correlation between seed coat color, flavonoids, and food nutrition needs to be further demonstrated in *Cucurbita maxima*. Whether the seed nutrition of yellow seed coat is significantly different from that of white seed coat also provides some new research prospects for variety breeding.

Flavonoids are one of the largest families of polyphenols, with complicated structural and functional diversity [56]. It is derived from phenylalanine and malonyl-coenzyme A (CoA) [57] and the C6-C3-C6 carbon bridge can be further divided by the nature of C3 element, accordingly producing nine major subgroups including chalcones, aurones, isoflavonoids, flavones, flavonols, anthocyanins, condensed tannins, and phlobaphene pigments [57]. Alanine and phenylalanine metabolism in the seed embryo of *Brassica napus* was found to be related to the embryonal control of seed coat color [58]. In *Brassica* species, yellow seed coat accessions exhibit a significantly thinner seed coat than that in black seed coat accessions and displayed a lower hull proportion [58]. Besides the major branch pathways of flavonoid biosynthesis, lignin is another branch of the phenylpropanoid pathway and participates in the formation of secondarily thickened cell walls [54]. In this study, a serial of metabolisms involved in flavonoids pathway including lignin, soluble PA, and insoluble PA displayed different distribution patterns in white and yellow seed coat accessions in *Cucurbita maxima*. Meanwhile, the secondary cell wall thickening of S-layers and the width of seed coat in Agol were obvious thinner than that in yellow seed coat accession Wuminglv (Figure 2). It was suggested that the mechanism of seed coat color determination and secondary cell wall thickening in *Cucurbita maxima* is associated with flavonoid metabolism and are similar to those in *Brassica* and *Glycine* species.

As oxidization compounds of flavonoid, PAs are thought to be the main pigment of seed coat in Arabidopsis, and it exists in the endothelial layer of the inner integument in seed coat [59,60]. In Arabidopsis, PAs synthesis is accompanied by seed coat development, cell differentiation, and seed maturation. In this study, flavonoid compounds in seed coat layer were determined by histochemical analysis during the seed development. At the beginning of pollination, the seed coat color of Wuminglv and Agol were both white. At 21~28 DPA, PAs were accumulated at the H layer and S layer accompanied by a secondary cell wall thickening at S layer, and the seed coat color turned yellow in Wuminglv. The pigment accumulation and the secondary cell structures of seed coat layer are inseparable [16,24], indicating that *WSC* may displayed a multiple function in seed coat development. Meanwhile, the development of seed coat in *Cucurbita maxima* possesses similar characteistics typically associated with that in *Cucurbita pepo*, indicating the regulatory of seed coat development displays a common pattern in *Cucurbita* species. 

In Arabidopsis and many crops, most of the phenotype of light seed coat color displayed recessive and maternal inheritance against dark seed coat color [61]. However, there are quantitative characters that affect seed coat color and some QTLs that have been identified [30,62,63]. In watermelon, the light cream color was recessive to the brown or red seed coat color, and any of these seed coat colors was recessive to the black color independently [31]. Some of the QTLs and candidate genes have been identified [7,30,32]. In this study, genetic analysis of six-generation population and F_2_ segregating population derived from different genetic background revealed that the phenotype of seed coat color was a recessive trait controlled by a single gene (Table 2 and Supplement Table S2). In the DMACA staining of 76 accessions, although all the accessions of white seed coat remain white or displayed a little pink, the degree of coloration of yellow seed coat ranged from dark red to light red, indicating that there may be some modification loci associated with pigment content, pigment deposition, or layer structure of the seed coat in *Cucurbita maxima*.

From the analysis of map-based cloning by F_2_ segregating population, *CmaCh15G005270* encoding R2 MYB transcription factor was predicted to be the candidate gene of white seed coat in *Cucurbita maxima*. In Arabidopsis, the homolog of *CmaCh15G005270* was *AtDIV1* (*DIVARICATA 1*) and *AtDIV2*. It is most similar to *LeMYBI* from tomato [52]. Interestingly, loss-of-function mutant *div2* was tolerant to salt stress and sensitivity to ABA during seed germination. *AtDIV2* played a negative role in salt stress and was required in ABA signal pathway [49]. *DIV* was studied in the ventral petal of *Antirrhinum majus* flowers [51,64]. An R2R3 MYB transcription factor AmMYBML1 (*Antirrhinum majus* MYB MIXTA LIKE 1) very similar to MIXTA was regulated by DIV in association with the B-function proteins MADS-domain transcription factors and control the specification of cell types in the ventral petal [64]. AmMYBML was the orthologous to MIXTA, while AmMYBML2 and AmMYBML3 were the orthologous to PhMYB1, AtMYB16, and AtMYB106. These R2R3-MYBs are not only involved in seed coat color formation and pigment accumulation, but also in other complicated physiological and biochemical regulation processes.

Sequence analysis revealed four main Insertion-Deletions of DNA sequences in the promoter regions. As displayed in 54 accessions, all the alleles exhibiting Insertion-Deletions in the promoter of *CmaCh15G005270* displayed a strong linkage with the phenotype of white or yellow seed coat. In many plants, it was an efficient way to regulate gene transcription through sequence variation of promoter. In *Sesamum indicum*, an AACACACAC-motif in single and duplicated copy in different germplasms displayed strong association with root biomass. It can be recognized by SiMYB181 in 5′-UTR of *Big root biomass* (*BRB*) and the loci have been selected by modern breeding [65]. In fruit quality trait studies, R2R3-MYB10 transcription factor were involved in anthocyanin biosynthesis in the color determination in peel and flesh and displayed a general regulatory role in the flavonoid/phenylpropanoid pathway during the ripening of fruits in *Fragaria* × *ananassa* (strawberry) [66], *Malus domestica* (apple) [67,68,69], *Pyrus pyrifolia* (pear) [70,71], *Prunus persica* (peach) [72], and other fruits. In the fruit of *Prunus persica*, PpMYB10.1 is the major regulator of anthocyanin accumulation in red-skinned fruit, and the allelic types of MYB10.1 displayed a close relationship with the intensity of red skin coloration [73]. In the leaves of *Prunus persica*, *PpMYB10.4* were identified in anthocyanin accumulation and 18 single nucleotide polymorphisms (SNP) and a 3-bp InDel were identified within a 2.0-kb region upstream the starting codon [72]. In *Malus domestica*, five direct tandem repeats of a 23-bp sequence forming a minisatellite-like structure contributed to an allelic rearrangement in the region of promoter and lead to ectopic accumulation of anthocyanins in the flesh and leaves of the plant [69]. In *Fragaria*, a CACTA-like transposon insertion in the *MYB10-2* promoter of red-fleshed accessions was associated with enhanced expression of *MYB10* and responsible for enhanced anthocyanin biosynthesis [74]. Recent studies on *MYB10-D* in synthetic hexaploidy wheat revealed that a 2.4 Mb presence-absence variation (PAV) region was involved in the flavonoid and ABA biosynthesis pathways, accordingly, the seed color of *MYB10-D* overexpression lines was red and that in the wild type was white [75]. The sequence variation of promoter region in MYB transcription has been reported to contribute to pigment deposition. In many vegetable crops, R2R3-MYBs such as MYB2, MYB75/PAP1, and PAP2 were involved in the anthocyanin regulatory in eggplant, radish, lablab pods, kohlrabi, and cabbages, not only in seed coat [26,76,77]. Therefore, the MYBs would be suitable for metabolic genetic engineering for improvement of fruit quality and vegetable nutrients [78].

MBW complex has been studied including R2R3-MYB, bHLH, and WD40 complex [59]. In *Phaseolus vulgaris* (Common bean), an R2R3 MYB was associated with seed coat darkening and involved in PAs oxidation and accumulation in the seed coat [79]. LACCASE family in Arabidopsis was involved in oxidative lignin polymerization and it was downstream of MYB46 and MYB58 [54]. A basic helix-loop-helix (bHLH) Noemi transcription factor controls the pigments. Lack of anthocyanins in young leaves and flowers is also associated with a lack of proanthocyanidins in seeds and, most notably, with an extreme reduction in fruit acidity [80,81,82].

Some components of regulation are upstream of MBW complex and are involved in the cell layer differentiation. The ovule identity factor SEEDSTICK (STK, a MADS transcription factor) displayed a connection between cell fate determination, seed coat development, and secondary metabolism as it controls PAs accumulation in the inner layer of seed coat by regulating the expression of *BANYULS/ANTHOCYANIDIN REDUCTASE* (*BAN*) though H3K9ac chromatin modification [83,84]. *TESTA8* and *GLABRA3* composited of WD40-bHLH-MYB complex are also downstream of *STK* [84,85]. In the promoter of *Ruby* gene, T-to-C transition on TATA and CAAT boxes was identified to determine the efficiency of the expression [80,81]. Cyanidin 3-(6′-malonyl)-β-glucoside and cyanidin 3-glucoside, accounting for most of the anthocyanin biosynthesis, were repressed in *ruby* accessions. Whether *WSC* directly participates in the formation of MBW complex like other R2R3-MYBs or indirectly regulates flavonoid synthesis by regulating downstream genes involved in MBW complex indirectly is still being studied.

The RNA-seq data displayed many genes involved in phenylpropanoid metabolism pathway and its downstream anthocyanin and flavonoid pathway. Meanwhile, RNA-seq data and qRT-PCR showed that *WSC* played a positive role in yellow seed coat formation. The expression pattern of *PAL*, *C4H*, *4CL*, *CHI*, and *DFR* were extremely different in Wuminglv and Agol at the stage of seed coat color formation. It can be confirmed that the color and structure of seed coat are closely related and affected by the same regulatory pathway. In this metabolic network, we have not only discovered genes related to pigment accumulation, but also some genes related to lignin metabolism. The target genes downstream of *CmaCh15G005270* are to be further identified.

## 4. Materials and Methods

### 4.1. Plant Materials

Inbred accessions Wuminglv (yellow seed coat color, P_1_), Agol (white seed coat color, P_2_), Lizimanhui (yellow seed coat color, P_3_), and Diaogua (white seed coat color, P_4_) derived from the Institute of Vegetables and Flowers, Chinese Academy of Agricultural Sciences (Beijing, China) were used as the parents. F_1_ was obtained through the hybridization of Wuminglv as female parents and Agol as male parents. A six-generation population including P_1_, P_2_, F_1_, F_2_, BC_1_, and BC_1_ was constructed by cross and self-pollination in Wuminglv and Agol. The 2798 plants in F_2_ segregating population derived from Wuminglv and Agol were used for the fine mapping. The 222 plants in F_2_ segregating population and RILs derived from Lizimanhui and Diaogua and 54 inbreds displayed different seed coat color were used to examine the accuracy of molecular markers.

### 4.2. Phenotypic Characterization

The seed coat color was categorized as two types by visual observation directly: the white seed type, or the yellow seed type. Due to the complexity of color concept, the L*, a*, and b* values were obtained by means of chromometer (HP-200, Hanpu, Changzhou), and the comprehensive chromaticity E was obtained by using the formula to carry out the quantitative comparison of seed coat color as E = [(L*)^2^ + (a*)^2^ + (b*)^2^]^1/2^.

### 4.3. Histochemical Analysis

Female flowers with conspicuous ovary were artificially pollinated and the date was recorded as 0 DPA (Day post anthesis). Three young fruits were harvested at 0, 7, 14, 21, 28, 35, and 42 DPA. The seeds were immediately collected and embedded in FAA solution at 4 °C (40% formaldehyde/70% glacial acetic acid/50% alcohol = 5:5:90). Paraffin sections were processed in Epsilon Company (Beijing, China) and toluidine blue was used for staining. The paraffin section of seed coat was observed by microscope Olympus BX51 (Tokyo, Japan). The width and length of each cell layer of the seed coat were measured by ImageJ.

### 4.4. DNA and RNA Extraction

Young leaf samples of P_1_, P_2_, F_1_, and all of the F_2_ plants with unique number were collected and frozen in liquid nitrogen. DNA extraction was performed as described by Xiang et al. [42]. For RNA extraction, the sample of seed coat were collected after removing the pulp in different development stages (0, 7, 14, 21, 28, 35, 42 DPA). Different tissues of older root, stem, young leaf, petals of anthesis, and ripening flesh were collected. RNA extraction was processed by MiniBEST Universal RNA Extraction Kit (TaKaRa, Japan, 9769).

### 4.5. Genetic Analysis

A six-generation population (derived from Wuminglv and Agol including P_1_, P_2_, F_1_, F_2_, BC_1_, BC_2_) and another F_2_ segregating population (derived from Lizimanhui and Diaogua and including P_3_, P_4_, F_1_, F_2_) were used to study the genetic analysis of seed coat color. The Chi-square test of fit was performed against the expected 3:1 segregation ratio in the F_2_ segregating population.

### 4.6. Map-Based Cloning of wsc Gene

The Whole genome sequencing was conductded in Wuminglv and Agol to develop the InDel markers for primary mapping (Biomarker Techonology Company, Beijing, China). Three hundred and twenty-four pairs of primers evenly distributed on each chromosome were designed by Primer 3.0 (http://bioinfo.ut.ee/primer3-0.4.0/, accessed on 6 February 2021) followed the reference genome (http://cucurbitgenomics.org/, accessed on 6 February 2021). The PCR program was conducted as follows: step 1, 95 °C 3 min; step 2, 95 °C 15 s, 54~56 °C 15 s, 72 °C 30 s, repeat 35 cycles; step 3, 72 °C 5 min. The DNA templates were amplified by high-fidelity Polymerase (P115, Vazyme, Nanjing, China) and the polymorphism of PCR products was determined by 8% Polyacrylamide gel electrophoresis (PAGE) in P_1_, P_2_, and F_1_. For fine mapping, DNA templates of 2798 F_2_ plants were amplified with primary flanking markers S1517 and S1528, followed by 17 InDel markers and nine dCAPS markers designed in the 315.4-kb candidate region for recombinants screening. The phenotype of seed coat color was confirmed in recombinants and the homozygous were selected to confirm the final candidate region.

### 4.7. Candidate Gene Prediction and Validation of Molecular Marker

The full lengths of four genes in the candidate region were amplified and sequenced from Wuminglv and Agol. The promoter region of candidate gene was amplified in 54 inbred accessions and sequenced in Sangon Biotech (Shanghai, China). The flanking InDel marker S1548 was used for validation of molecular markers in 54 inbred accessions and 222 lines of F_2_ segregating population and RILs derived from P_3_ and P_4_.

### 4.8. RNA-Seq and Spatiotemporal Expression Analysis

The samples of seed coat were collected after removing the pulp in different development stages (0, 7, 14, 21, 28, 35, 42 DPA). RNA-seq was conducted on Illumina platform with three replicates for each sample in Biomarker Techonology Company (Beijing, China). The sequencing Reads were compared to the reference genome using TopHat2 [86]. The transcriptome assemblies were performed by Cufflflinks [87]. Then, according to the expression level of genes in seed coat samples in different periods, DESeq software was used for differential expression analysis, and differentially expressed genes were screened through indicators FDR = 0.01 and FC = 2 [88]. Finally, the annotation information of each differentially expressed gene set was extracted for bioinformatics analysis.

For gene expression analysis, ChamQ Universal SYBR qPCR Master Mix (Q711, Vazyme, Nanjing, China) was used and the expression of candidate genes was determined by quantitative Real-time PCR (Bio-Rad, C1000 Touch, Foster City, CA, USA). The data was analyzed by Bio-Red CFX Manager (Version 3.1, Bio-Rad).

### 4.9. DMACA Staining and PA Determination

DMACA staining and PA determination were followed as in Pang et al. [23]. Total soluble PA content was calculated spectrophotometrically after reaction with DMACA reagent (0.2% wt/vol DMACA in methanol-3N HCl) at 640 nm by Spectrophotometer (Unico, UV/VIS 2802PC, Princeton, NJ, USA with (+)-catechin (Solarbio, 154-23-4, Beijing, China) as standard. For total insoluble PA analysis, Absorbance values were converted into PA equivalents using a standard curve of procyanidin B1 (4852-22-6, MYM, Beijing, China).

### 4.10. Quantitative Determination for Lignin

Quantitative determination for lignin was followed as in [89]. Aliquots of 20 mg of seed coat (six replicates per individual sample) of Wuminglv and Agol were mixed thiogylcolic acid (MYM, Beijing, China) and the standard lignin (Biotapped, Beijing, China) was used to generate the calibration curves.

## Figures and Tables

**Table 1:** Seed coat color values of parents and segregating populations obtained by chromometer.

	L*	a*	b*	E
P_1_ (yellow)	68.89 ± 3.40	12.45 ± 6.02	25.14 ± 6.68	75.32 ± 3.95
P_2_ (white)	86.71 ± 6.08	0.98 ± 0.96	4.44 ± 3.05	86.89 ± 5.90
F_1_ (P_1_ × P_2_) (yellow)	66.13 ± 4.69	11.46 ± 2.12	19.74 ± 5.41	70.82 ± 5.03
F_1_′ (P_1_ × P_2_) (yellow)	67.03 ± 4.08	11.13 ± 2.58	19.54 ± 5.04	70.83 ± 5.41
F_2_ (separation)	59.63~94.71	0.04~17.28	1.67~30.46	64.53~94.80
BC_1_ (yellow)	68.71 ± 6.62	11.94 ± 4.40	21.43 ± 5.24	72.41 ± 6.23
BC_2_ (separation)	60.89~96.27	0.01~15.05	0.87~21.83	64.17~96.27
P_3_ (yellow)	65.35 ± 4.96	7.59 ± 3.66	19.39 ± 3.46	68.89 ± 5.28
P_4_ (white)	82.67 ± 3.07	3.57 ± 1.58	15.13 ± 4.53	83.79 ± 2.62
F_1_ (P_3_ × P_4_) (yellow)	64.66 ± 4.29	13.61 ± 2.45	21.95 ± 2.59	69.58 ± 4.02
F_1_’ (P_3_ × P_4_) (yellow)	64.28 ± 5.11	13.09 ± 2.44	23.82 ± 2.42	69.48 ± 4.81

E = [(L*)^2^ + (a*)^2^ + (b*)^2^]^1/2^.

**Table 2:** The segregation proportion of yellow seed coat and white seed coat in P_1_, P_2_, F_1_, F_1_′, F_2_, BC_1_, and BC_2_.

Population	Number of Plants			Expected Ratio	Chi Square Test
	Yellow Seed Coat	White Seed Coat	Total		
P_1_	5	0	5		
P_2_	0	6	6		
F_1_	13	0	13		
F_1_’	13	0	13		
F_2_	282	95	377	3:1	χc^2^ = 0.002 < 0.05^2^ = 3.84
BC_1_	109	0	109		
BC_2_	65	56	121	1:1	χc^2^ = 0.17 < 0.05^2^ = 3.84

**Table 3:** Annotation and homologs of candidate genes in Arabidopsis thaliana.

Gene ID	Location in Chr.15	Gene Function Annotation	Homologs in At	Gene Description in TAIR10
CmaCh15G005220	2407955~2413296	Vesicle-associated membrane protein 727	AT3G54300	Synaptobrevin-like protein family
CmaCh15G005230	2413306~2414285	Unknown protein	–	–
CmaCh15G005240	2415047~2420995	AMMECR1 family	AT2G38710	AMMECR1 family
CmaCh15G005250	2421828~2424505	Multiple myeloma tumor-associated protein, putative	AT3G52220	Leukocyte immunoglobulin-like receptor family protein
CmaCh15G005260	2424963~2427834	F1F0-ATPase inhibitor protein	AT5G04750	F1F0-ATPase inhibitor protein
CmaCh15G005270	2428976~2431637	MYB transcription factor	AT5G04760	R-R-type MYB protein which plays negative roles in salt stress and is required for ABA signaling in *Arabidopsis*
CmaCh15G005280	2441111~2445124	Cationic amino acid transporter 7	AT5G04770	Cationic amino acid transporter (CAT) subfamily of amino acid polyamine choline transporters
CmaCh15G005290	2449424~2462027	Pentatricopeptide repeat-containing protein	AT5G04780	Pentatricopeptide repeat (PPR) superfamily protein
CmaCh15G005300	2463445~2466291	Double Clp-N motif-containing P-loop nucleoside triphosphate hydrolases superfamily protein, putative	AT3G52490	SMAX1 and SMAX1-like protein that has weak similarity to *AtHSP101*

## Supplementary Materials

Supplementary materials can be found at https://www.mdpi.com/1422-0067/22/6/2972/s1.
