# Supplementary material for: Genetic Mapping and Identification of the Candidate Gene for White Seed Coat in Cucurbita maxima
Source: Int J Mol Sci. 2021 Mar 15;22(6):2972. doi: 10.3390/ijms22062972 (PMC8000038; doi:10.3390/ijms22062972)
Supplement: Supplementary file 1 [file ijms-22-02972-s001.pdf]

Supplementary Materials

Genetic Mapping and Identification of the Candidate Gene for White Seed Coat in *Cucurbita maxima*

Shi Yuzi, Zhang Meng, Shu Qin, Ma Wei, Sun Tingzhen, Xiang Chenggang, Wang Changlin * and Duan Ying *

Key Laboratory of Biology and Genetic Improvement of Horticultural Crops of Ministry of Agriculture and Rural Affairs, Institute of Vegetables and Flowers, Chinese Academy of Agricultural Sciences, Beijing 100081, China; syz96421@yeah.net (S.Y.); 3292723551@qq.com (Z.M.); shuqin-fine@163.com (S.Q.); 15930279531@163.com (M.W.); s_tingzhen@foxmail.com (S.T.); z0501080535@126.com (X.C.)

* Correspondence: wangchanglin@caas.cn (W.C.); duanying@caas.cn (D.Y.); Tel.:86-10-8210-9547

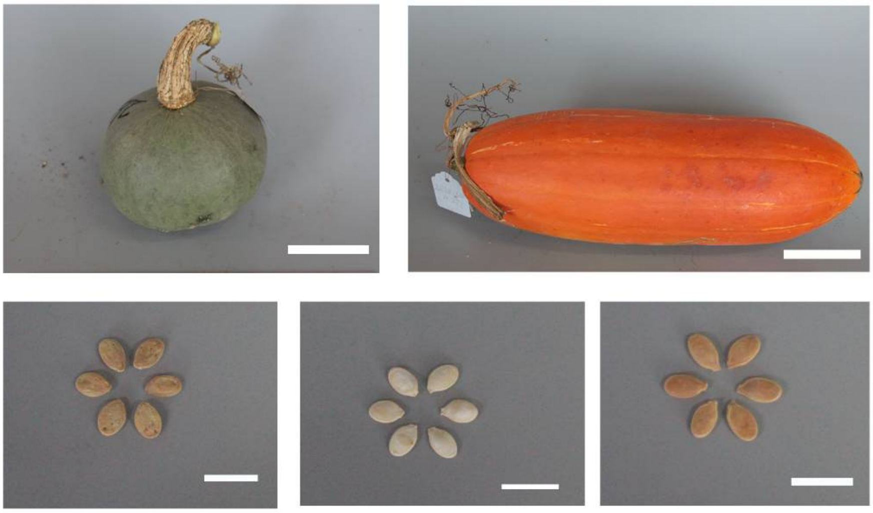

Supplement Figure S1. Phenotype of mature fruit and seed coat color of Lizimanhui.

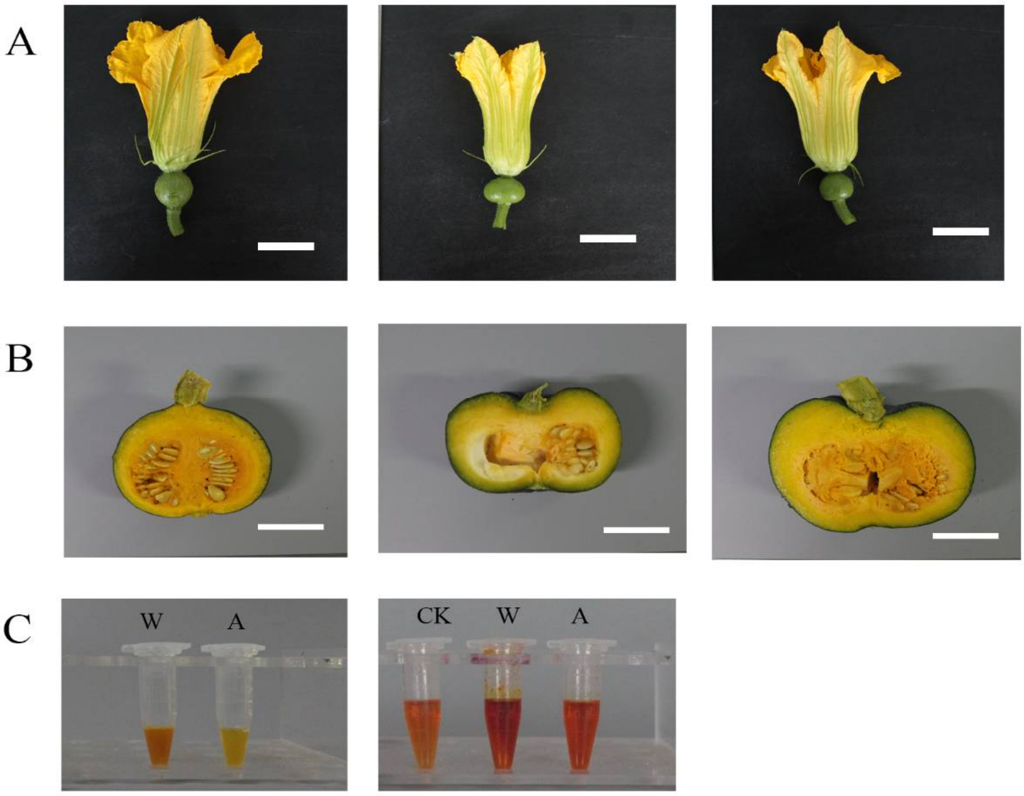

Supplement Figure S2. The phenotype of flowers, flesh and DMACA staining in extraction of flesh in Wuminglv and Agol.

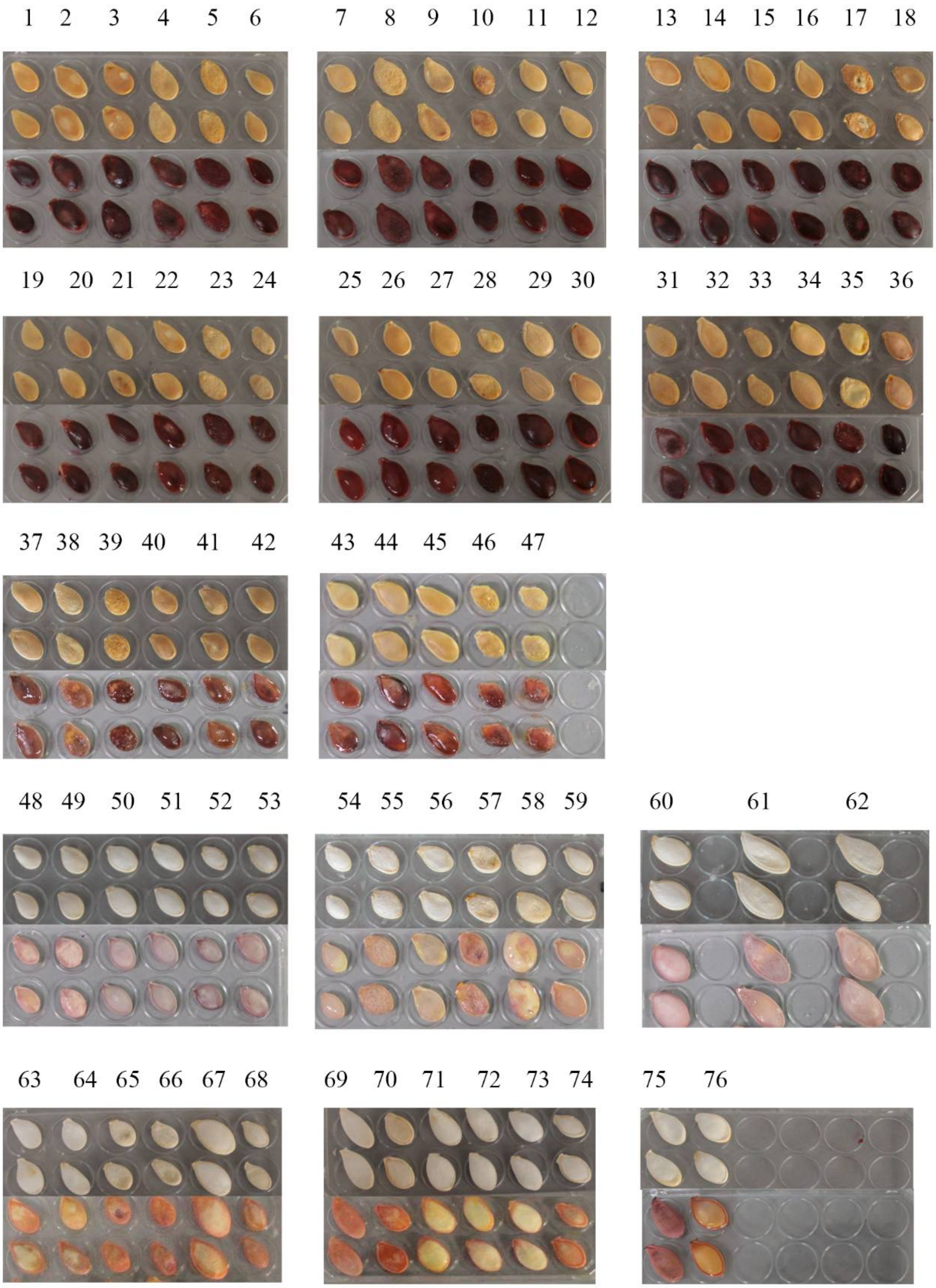

Supplement Figure S3. DMACA staining in 76 inbred accessions with different genetic backgrounds.

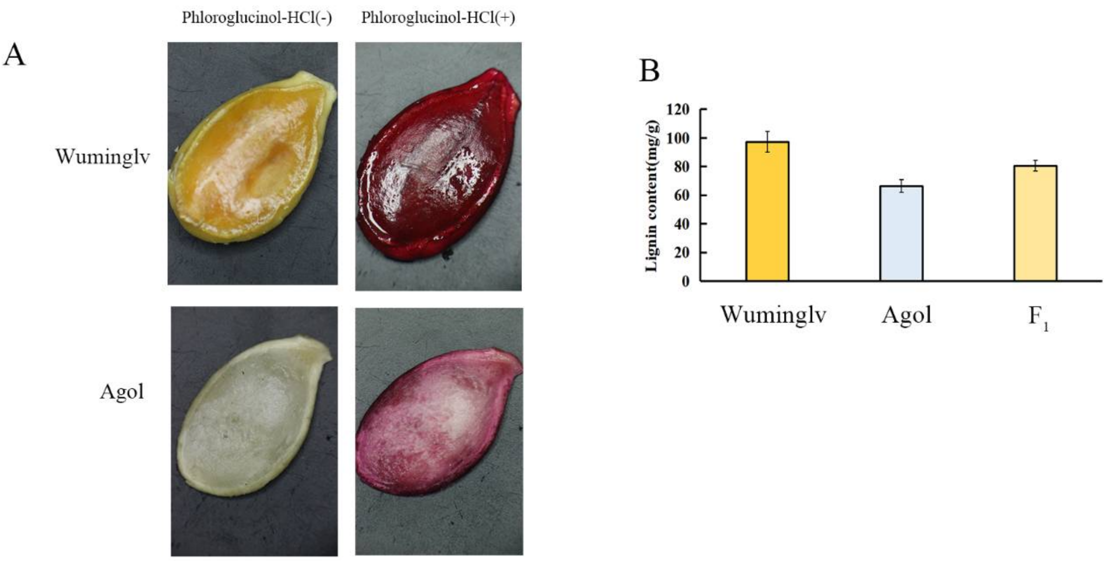

Supplement Figure S4. Lignin determination in seed coat.

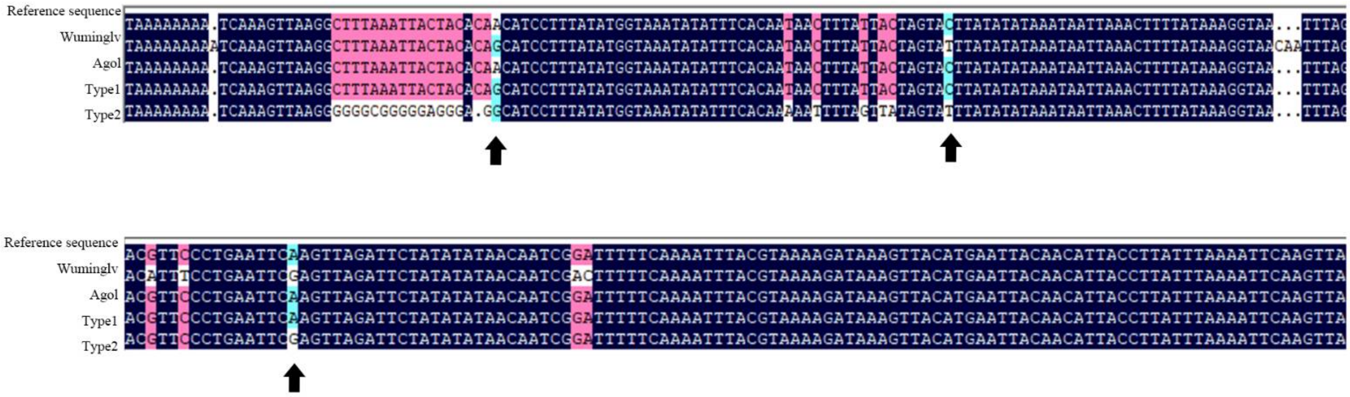

Supplement Figure S5. Three types of sequence variation in the promoter region.

Supplement Table S1. Color determination of F_2 population for primary mapping.

No.	Phenotype	L*	E	No.	Phenotype	L*	E	No.	Phenotype	L*	E	No.	Phenotype	L*	E	No.	Phenotype	L*	E
1	N	68.03	73.40	25	N	73.96	80.44	49	N	66.36	71.13	73	W	91.59	91.78	97	N	72.44	78.30
2	N	68.48	75.37	26	N	67.03	71.54	50	N	72.47	76.66	74	N	62.11	69.51	98	N	66.47	71.49
3	N	74.27	78.49	27	W	84.97	85.72	51	N	68.15	73.49	75	W	92.68	92.70	99	W	89.80	90.18
4	N	71.58	76.51	28	N	69.23	75.20	52	N	73.98	79.40	76	W	85.50	86.17	100	N	68.62	72.22
5	W	90.18	90.27	29	N	72.11	76.87	53	N	67.90	72.82	77	W	84.33	85.26	101	N	63.86	67.44
6	N	68.26	72.04	30	N	68.15	72.16	54	N	67.48	74.21	78	N	69.77	74.34	102	N	68.28	73.75
7	N	66.02	70.90	31	N	68.42	74.26	55	N	69.88	74.63	79	N	66.34	71.26	103	W	85.89	86.62
8	N	65.95	70.91	32	N	73.71	78.35	56	N	67.41	72.64	80	N	66.26	73.03	104	N	67.41	72.71
9	N	64.52	68.78	33	N	67.97	72.42	57	W	93.37	93.39	81	N	70.95	77.19	105	W	93.84	93.87
10	N	71.63	76.46	34	N	68.74	75.20	58	N	68.73	73.54	82	N	61.68	66.21	106	N	68.42	72.09
11	W	92.14	92.17	35	W	93.65	93.87	59	N	68.63	74.25	83	N	65.43	71.95	107	N	67.05	72.94
12	N	67.40	72.22	36	N	68.37	73.10	60	W	94.24	94.38	84	N	68.58	73.28	108	N	69.00	73.48
13	W	87.94	88.41	37	N	67.77	74.81	61	N	64.75	69.26	85	W	85.81	86.43	109	N	64.59	70.94
14	W	88.29	88.67	38	N	71.34	76.37	62	N	70.54	74.41	86	N	70.21	74.82	110	N	68.69	73.93
15	N	72.25	77.47	39	N	69.08	73.12	63	W	85.60	86.37	87	W	85.61	86.64	111	W	93.80	93.83
16	N	67.73	73.12	40	N	68.04	74.83	64	N	66.47	71.90	88	N	61.27	64.53	112	W	87.19	87.77
17	N	67.43	72.24	41	N	70.93	76.22	65	W	87.15	87.68	89	N	69.08	74.26	113	N	66.77	71.11
18	N	67.93	74.00	42	N	72.42	79.18	66	N	70.05	74.13	90	N	62.08	66.52	114	N	68.02	73.55
19	W	87.70	88.33	43	N	66.78	72.54	67	N	71.51	77.33	91	N	64.02	71.11	115	N	72.82	77.02
20	W	94.71	94.80	44	N	73.12	77.02	68	N	67.50	72.45	92	W	85.75	86.10	116	N	68.43	73.78
21	N	65.65	71.17	45	W	88.32	88.77	69	N	66.51	71.42	93	N	66.50	71.25	117	N	59.63	66.18
22	N	71.04	75.78	46	N	69.10	73.69	70	N	71.66	77.46	94	N	65.99	71.84	118	W	91.55	91.60
23	N	60.42	67.08	47	W	88.80	89.36	71	N	69.97	75.00	95	W	93.49	93.54	119	N	74.30	80.51
24	W	87.94	88.25	48	N	70.80	76.24	72	W	90.40	90.60	96	N	71.20	76.16	120	W	83.27	83.90

Supplement Table S2. The segregation proportion of yellow seedcoat and white seed coat in Lizimanhui(P_3), Diaogua (P_4), F_1, F_1', and F_2 segregating population.

Population	Number of Plants			Expected Ratio	^{2} Chi Square Test
	Yellow Seed Coat	White Seed Coat	Total		
P_3	13	0	13		
P_4	0	14	14		
F_1	25	0	25		
F_1'	26	0	26		
F_2	159	53	212	3:1	$_c{}^2 = 0$ $_{0.05}{}^2 = 3.84$

Supplement Table S3. Markers designed in map-based cloning and qRT-PCR.

Mapping Maker	Sequence 5'–3'	Position	Type	Restriction Endonuclease
SD-1F	CCCCAAAAAAGCTAAGAAGGAGAGG	2383171	dCAPs	HaeIII
SD-1R	TCCTTCCCATACAGATGACCTT			
SD-2F	TATTTTGGATTAATTGATTTTAAAT	2385945	dCAPs	VspI
SD-2R	CAAGATGATCAGGGTGTGGA			
SD-3F-2	AAGCTTCTCTGAAGTATTGGATCTC	2395953	dCAPs	XhoI
SD-3R	TGCAATTTCCACTGCATCTC			
SD-4F	AATGAAAAATCACTTTCCGATTGGC	2408617	dCAPs	HaeIII
SD-4R	TGCATTGCTTAGACGGAGTG			
SD-5F	GACAGACTATGGTTGCAAACTTCTA	2418406	dCAPs	XbaI
SD-5R	AACTTTGCCCAAATCAGTTGAC			
SD-6F	TTCGCCATTTTAAGTCTTCTTCTCG	2441150	dCAPs	TaqI
SD-6R	GTGGACACCGGAAGTGTACC			
SD-7F	TTCGAATCTCAAACAATGTTCTGGC	2456461	dCAPs	HaeIII
SD-7R	CCAATATGAACCATGAATCCAG			
S1517-F	CACTGAAAGGAGGGACAAGC	2357909	InDel	
S1517-R	CCTTGGCTGTGATCATTGTG			
S1518-F	TCTGCATTGCCATTAGGTTG	2549380	InDel	
S1518-R	TTTAACCTCGGGAACATCCA			
S1520-F	ACCCAATTGTTCGGGTTTTT	2446509	InDel	
S1520-R	CGTGGCATGAGTGGAAGTC			
S1522-F	CTCGAGCAATGTTCCCTTGT	2469039	InDel	
S1522-R	CGTGTTTGACCGGTAAGTTCA			
S1523-F	TTGAATCGTGTGTGAAGAACG	2494771	InDel	
S1523-R	TTCCGGTTTGGGTTTTGTTA			
S1524-F	ATTGTGCCTTGGTTGCATTT	2512540	InDel	
S1524-R	TGCAAATTCGTTTGCTTAGG			
s1527-F	ATGATACCCGTCCACACTGG	2606967	InDel	
s1527-R	AATTTGATCGACACCCTGCT			
s1528-F	GTATGGGCAAGGGTGTCAAG	2673260	InDel	
s1528-R	CAACTCGAACTGAACCAGACA			
S1532-F	GGATTTCATGGTCCCAAATG	2472668	InDel	
S1532-R	CGACGGCACTCATACATCAC			
s1534-F	AGGCCAACTTTTGTTGATGC	2475233	InDel	
s1534-R	TTGACGTGAGGGTGAGAATG			

Mapping Maker	Sequence 5'–3'	Position	Type	Restriction Endonuclease
s1536-F	CATTCATGCATGGACTTGGA	2539229	InDel	
s1536-R	ATCGTGCATAAACCAGACCA			
s1538-F	CAAGGACACTAGGCCCTGAA	2367263	InDel	
s1538-R	TTTGTTCTCCTTCCCAATCG			
s1539-F	GATGCAGCATGCAGTACTTACC	2372904	InDel	
s1539-R	TGGGAAGCTTGACAACACTG			
s1540-F	TTATCCATCGAGCATGTTGC	2374362	InDel	
s1540-R	AATACACACCGACACCACCA			
s1543-F	TTCCAAACAACATCAAGTGGAC	2446818	InDel	
s1543-R	TCACGTTAGTCATCGTGTCAA			
s1544-F	TCGTTATCCAATGAATTGACATT	2447732	InDel	
s1544-R	ATGTCTCAACCCAACCCAAC			
s1546-F	ACAACCTCGAGCAATGTTCC	2468979	InDel	
s1546-R	CGTGTTTGACCGGTAAGTTC			

qRT-PCR maker information

qRT-PCR Maker	Sequence 5'–3'
Q-5270-F	GCC GGA ATG GGA CTC TTC TGG A
Q-5270-R	GAT GCT CCT CCA ATC GCC CTT G
Q-CHI-F	CCG GCC TCT GCT AAG ACT CTA TTC C
Q-CHI-R	CCA CTT ACC GGC AAG CAA TGG C
Q-PAL-F	GCT ATG GTG TCA CCA CCG GTT TC
Q-PAL-R	ATT CCG TGT CGC TGC CCA A
Q-C4H-F	CAA GGA CTA TTT CGT CGA GGA GAG GA
Q-C4H-R	CAG TGA TCG GAA CAT CAG CTC CAA GTA C
Q-4CL-2F	AGC CGA GCT TGA GTC GCT TCT TA
Q-4CL-2R	CTT CCT CTG TTA TTG CGC CGC C
Q-DFR-F	GAG CCT TCT CGA GAA TGG CTA CTC T
Q-DFR-R	CGG GTC GTC TAG ATC AGC ATG GT
Q-Peroxidase-F	CCA ACT CTC CCC AAC ATT GTC CG
Q-Peroxidase-R	GCT CAC TGA TTA TGC CCG GAG C
Q-CCR4-F	GCA GCT ATT ATG GGC TGT GAT GGT G
Q-CCR4-R	CGA TGG ATG ATG TGA ACA CCA CTC GTC
Q-P450-F	GAC CCT TCT TGC GAG GTT ATT TGA ACA AGT G
Q-P450-R	ACT GAG CGT CTA TGA TAT GAT CCA TAG CAC AAC
Q-4CL-F	GAG CTT CTC TTC CTA GGA ATC AGC CC
Q-4CL-R	CGT CGA CGT AAC CTA TGT CGC C
Q-CAD-F	ATG TGT ATC TCG ACG GCC GAC C
Q-CAD-R	CCT CTT AGC CCA CTC TGT TTC AGT CC
Q-C4H-2F	GTT GGA TGC GCA ACA GAA GGG AG
Q-C4H-2R	GTC CGA GTA CAG TGT CGA GCT CA
Q-4CL-3F	CGA CGA GAT CTT CAT CGT CGA TCG AC
Q-4CL-3R	GCA GCC GGG ATC TCT CCA G

Supplement Table S4. Output statistics of RNA-seq sequencing data.

Samples	Clean Reads	Clean Bases	GC Content	% ≥ Q30
A0-1	22,396,797	6,711,603,654	45.69%	92.72%
A0-2	19,958,466	5,975,742,478	45.88%	91.76%
A0-3	19,620,559	5,877,179,176	45.82%	92.85%
A7-1	21,090,755	6,305,602,388	45.81%	92.78%
A7-2	25,630,791	7,664,938,890	45.71%	93.47%
A7-3	23,102,094	6,910,793,380	45.63%	93.10%
A14-1	28,949,564	8,637,191,840	45.29%	92.55%
A14-2	24,437,511	7,271,341,766	45.48%	92.35%
A14-3	25,667,247	7,653,236,676	45.57%	92.68%
A21-1	20,303,197	6,077,673,468	45.49%	92.04%
A21-2	28,704,246	8,598,629,892	45.85%	93.10%
A21-3	28,197,272	8,416,615,752	45.53%	92.67%
A28-1	22,089,870	6,619,234,430	45.60%	93.56%
A28-2	19,491,534	5,840,690,594	46.03%	92.68%
A28-3	21,288,863	6,376,913,928	45.09%	92.95%
A35-1	20,363,893	6,106,912,738	45.45%	93.86%
A35-2	23,150,598	6,922,455,694	45.63%	93.51%
A35-3	24,985,202	7,466,184,928	46.85%	93.45%
A42-1	21,966,185	6,477,942,300	45.41%	93.49%
A42-2	23,253,092	6,969,917,702	46.24%	92.60%
A42-3	26,278,500	7,854,552,730	45.63%	94.27%
W0-1	23,419,237	7,016,137,530	45.77%	91.96%
W0-2	35,956,189	10,736,907,094	45.57%	93.37%
W0-3	22,444,344	6,721,308,174	45.58%	92.26%
W7-1	20,394,319	6,097,382,316	45.63%	92.54%
W7-2	24,221,671	7,248,820,830	45.70%	93.27%
W7-3	22,417,132	6,710,914,836	45.62%	93.78%
W14-1	23,989,767	7,188,848,436	45.19%	94.08%
W14-2	20,942,644	6,231,117,038	45.73%	92.63%
W14-3	23,418,538	7,017,465,644	45.61%	93.91%
W21-1	32,411,815	9,687,868,946	44.96%	91.99%
W21-2	23,158,621	6,939,608,482	45.07%	92.21%
W21-3	38,365,062	11,404,234,246	44.94%	92.15%
W28-1	23,840,721	7,136,406,526	45.33%	91.85%
W28-2	22,357,710	6,688,011,076	45.00%	92.63%
W28-3	23,189,188	6,934,833,462	45.00%	91.63%
W35-1	22,751,807	6,810,446,138	45.11%	91.56%
W35-2	19,205,379	5,737,595,578	45.10%	91.05%
W35-3	32,064,444	9,501,167,902	45.28%	91.06%
W42-1	38,826,061	11,592,251,052	45.08%	90.57%
W42-2	31,864,150	9,535,642,038	45.02%	92.27%
W42-3	28,778,945	8,594,461,270	45.25%	92.53%

Supplement Table S5. Validation of molecular marker S1548.

No.	Name	Phenotype	Genotype	No.	Name	Phenotype	Genotype
1	241	Y	A	36	293	Y	A
2	242	W	a	37	295	Y	A
3	243	W	a	38	296	Y	A
4	244	W	a	39	300	W	a
5	245	W	a	40	302	W	a
6	246	W	a	41	306	W	a
7	247	W	a	42	308	W	a
8	250	W	a	43	310	Y	A
9	251	W	a	44	311	W	a
10	256	W	a	45	488	Y	A
11	257	W	a	46	489	Y	A
12	258	Y	A	47	492	W	a
13	261	Y	A	48	495	Y	A
14	262	Y	A	49	497	Y	A
15	263	W	a	50	504	W	a
16	264	W	a	51	505	W	a
17	266	Y	A	52	506	W	a
18	267	Y	A	53	508	Y	A
19	268	Y	A	54	521	W	a
20	269	W	a				
21	271	Y	A				
22	272	Y	A				
23	277	Y	A				
24	278	Y	A				
25	279	Y	A				
26	280	Y	A				
27	281	Y	A				
28	282	Y	A				
29	283	Y	A				
30	284	Y	A				
31	287	Y	A				
32	288	Y	A				
33	289	W	a				
34	291	Y	A				
35	292	Y	A				

No.	Origin	Phenotype	Genotype	No.	Origin	Phenotype	Genotype	No.	Origin	Phenotype	Genotype
1	F_2 (P_3×P_4)	W	a	36	F_2 (P_3×P_4)	W	a	78	P_3×P_4 RILs	W	a
2	F_2 (P_3×P_4)	W	a	37	F_2 (P_3×P_4)	W	a	79	P_3×P_4 RILs	W	a
3	F_2 (P_3×P_4)	W	a	38	F_2 (P_3×P_4)	W	a	80	P_3×P_4 RILs	W	a
4	F_2 (P_3×P_4)	W	a	39	F_2 (P_3×P_4)	W	a	81	P_3×P_4 RILs	W	a
5	F_2 (P_3×P_4)	W	a	40	F_2 (P_3×P_4)	W	a	82	P_3×P_4 RILs	W	a
6	F_2 (P_3×P_4)	W	a	41	F_2 (P_3×P_4)	W	a	83	P_3×P_4 RILs	W	a
7	F_2 (P_3×P_4)	W	a	42	F_2 (P_3×P_4)	W	a	84	P_3×P_4 RILs	W	a
8	F_2 (P_3×P_4)	W	a	43	F_2 (P_3×P_4)	W	a	85	P_3×P_4 RILs	W	a
9	F_2 (P_3×P_4)	W	a	44	F_2 (P_3×P_4)	W	a	86	P_3×P_4 RILs	W	a
10	F_2 (P_3×P_4)	W	a	45	F_2 (P_3×P_4)	W	a	87	P_3×P_4 RILs	W	a
11	F_2 (P_3×P_4)	W	a	46	F_2 (P_3×P_4)	W	a	88	P_3×P_4 RILs	W	a
12	F_2 (P_3×P_4)	W	a	47	F_2 (P_3×P_4)	W	a	89	P_3×P_4 RILs	W	a
13	F_2 (P_3×P_4)	W	a	48	F_2 (P_3×P_4)	W	a	90	P_3×P_4 RILs	W	a
14	F_2 (P_3×P_4)	W	a	49	F_2 (P_3×P_4)	W	a	91	P_3×P_4 RILs	W	a
15	F_2 (P_3×P_4)	W	a	50	F_2 (P_3×P_4)	W	a	92	P_3×P_4 RILs	W	a
16	F_2 (P_3×P_4)	W	a	51	F_2 (P_3×P_4)	W	a	93	P_3×P_4 RILs	W	a
17	F_2 (P_3×P_4)	W	a	52	F_2 (P_3×P_4)	W	a	94	P_3×P_4 RILs	W	a
18	F_2 (P_3×P_4)	W	a	53	F_2 (P_3×P_4)	W	a	95	P_3×P_4 RILs	W	a
19	F_2 (P_3×P_4)	W	a	54	F_2 (P_3×P_4)	W	a	96	P_3×P_4 RILs	W	a
20	F_2 (P_3×P_4)	W	a	55	F_2 (P_3×P_4)	W	a	97	P_3×P_4 RILs	W	a
21	F_2 (P_3×P_4)	W	a	56	F_2 (P_3×P_4)	W	a	98	P_3×P_4 RILs	W	a
22	F_2 (P_3×P_4)	W	a	57	F_2 (P_3×P_4)	W	a	99	P_3×P_4 RILs	W	a
23	F_2 (P_3×P_4)	W	a	58	F_2 (P_3×P_4)	W	a	100	P_3×P_4 RILs	W	a
24	F_2 (P_3×P_4)	W	a	59	F_2 (P_3×P_4)	W	a	101	P_3×P_4 RILs	W	a
25	F_2 (P_3×P_4)	W	a	60	F_2 (P_3×P_4)	W	a	102	P_3×P_4 RILs	W	a
26	F_2 (P_3×P_4)	W	a	61	F_2 (P_3×P_4)	W	a	103	P_3×P_4 RILs	W	a
27	F_2 (P_3×P_4)	W	a	62	F_2 (P_3×P_4)	W	a	104	P_3×P_4 RILs	W	a
28	F_2 (P_3×P_4)	W	a	70	P_3×P_4 RILs	W	a	105	P_3×P_4 RILs	W	a
29	F_2 (P_3×P_4)	W	a	71	P_3×P_4 RILs	W	a	106	P_3×P_4 RILs	W	a
30	F_2 (P_3×P_4)	W	a	72	P_3×P_4 RILs	W	a	107	P_3×P_4 RILs	W	a
31	F_2 (P_3×P_4)	W	a	73	P_3×P_4 RILs	W	a	108	P_3×P_4 RILs	W	a
32	F_2 (P_3×P_4)	W	a	74	P_3×P_4 RILs	W	a	109	P_3×P_4 RILs	W	a
33	F_2 (P_3×P_4)	W	a	75	P_3×P_4 RILs	W	a	110	P_3×P_4 RILs	W	a
34	F_2 (P_3×P_4)	W	a	76	P_3×P_4 RILs	W	a	111	P_3×P_4 RILs	W	a
35	F_2 (P_3×P_4)	W	a	77	P_3×P_4 RILs	W	a	112	P_3×P_4 RILs	W	a

No.	Origin	Pheno-type	Geno-type	No.	Origin	Pheno-type	Geno-type	No.	Origin	Pheno-type	Geno-type
113	P_3×P_4 RILs	W	a	150	P_3×P_4 RILs	W	a	188	P_3×P_4 RILs	Y	A
114	P_3×P_4 RILs	W	a	151	P_3×P_4 RILs	W	a	189	P_3×P_4 RILs	Y	A
115	P_3×P_4 RILs	W	a	152	P_3×P_4 RILs	W	a	190	P_3×P_4 RILs	Y	A
116	P_3×P_4 RILs	W	a	153	P_3×P_4 RILs	W	a	191	P_3×P_4 RILs	Y	A
117	P_3×P_4 RILs	W	a	154	P_3×P_4 RILs	W	a	192	P_3×P_4 RILs	Y	A
118	P_3×P_4 RILs	W	a	155	P_3×P_4 RILs	W	a	193	P_3×P_4 RILs	Y	A
119	P_3×P_4 RILs	W	a	156	P_3×P_4 RILs	W	a	194	P_3×P_4 RILs	Y	A
120	P_3×P_4 RILs	W	a	157	P_3×P_4 RILs	W	a	195	P_3×P_4 RILs	Y	A
121	P_3×P_4 RILs	W	a	158	P_3×P_4 RILs	W	a	196	P_3×P_4 RILs	Y	A
122	P_3×P_4 RILs	W	a	159	P_3×P_4 RILs	W	a	197	P_3×P_4 RILs	Y	A
123	P_3×P_4 RILs	W	a	160	P_3×P_4 RILs	W	a	198	P_3×P_4 RILs	Y	A
124	P_3×P_4 RILs	W	a	161	P_3×P_4 RILs	W	a	199	P_3×P_4 RILs	Y	A
125	P_3×P_4 RILs	W	a	162	P_3×P_4 RILs	W	a	200	P_3×P_4 RILs	Y	A
126	P_3×P_4 RILs	W	a	163	P_3×P_4 RILs	W	a	201	P_3×P_4 RILs	Y	A

127	P_3×P_4 RILs	W	a	164	P_3×P_4 RILs	W	a	202	P_3×P_4 RILs	Y	A
128	P_3×P_4 RILs	W	a	165	P_3×P_4 RILs	W	a	203	P_3×P_4 RILs	Y	A
129	P_3×P_4 RILs	W	a	166	P_3×P_4 RILs	W	a	204	P_3×P_4 RILs	Y	A
130	P_3×P_4 RILs	W	a	167	P_3×P_4 RILs	W	a	205	P_3×P_4 RILs	Y	A
131	P_3×P_4 RILs	W	a	169	P_3×P_4 RILs	Y	H	206	P_3×P_4 RILs	Y	A
132	P_3×P_4 RILs	W	a	170	P_3×P_4 RILs	Y	H	207	P_3×P_4 RILs	Y	A
133	P_3×P_4 RILs	W	a	171	P_3×P_4 RILs	Y	H	208	P_3×P_4 RILs	Y	A
134	P_3×P_4 RILs	W	a	172	P_3×P_4 RILs	Y	H	209	P_3×P_4 RILs	Y	A
135	P_3×P_4 RILs	W	a	173	P_3×P_4 RILs	Y	A	210	P_3×P_4 RILs	Y	A
136	P_3×P_4 RILs	W	a	174	P_3×P_4 RILs	Y	A	211	P_3×P_4 RILs	Y	A
137	P_3×P_4 RILs	W	a	175	P_3×P_4 RILs	Y	A	212	P_3×P_4 RILs	Y	A
138	P_3×P_4 RILs	W	a	176	P_3×P_4 RILs	Y	A	213	P_3×P_4 RILs	Y	A
139	P_3×P_4 RILs	W	a	177	P_3×P_4 RILs	Y	A	214	P_3×P_4 RILs	Y	A
140	P_3×P_4 RILs	W	a	178	P_3×P_4 RILs	Y	A	215	P_3×P_4 RILs	Y	A
141	P_3×P_4 RILs	W	a	179	P_3×P_4 RILs	Y	A	216	P_3×P_4 RILs	Y	A
142	P_3×P_4 RILs	W	a	180	P_3×P_4 RILs	Y	A	217	P_3×P_4 RILs	Y	A
143	P_3×P_4 RILs	W	a	181	P_3×P_4 RILs	Y	A	218	P_3×P_4 RILs	Y	A
144	P_3×P_4 RILs	W	a	182	P_3×P_4 RILs	Y	A	219	P_3×P_4 RILs	Y	A
145	P_3×P_4 RILs	W	a	183	P_3×P_4 RILs	Y	A	220	P_3×P_4 RILs	Y	A
146	P_3×P_4 RILs	W	a	184	P_3×P_4 RILs	Y	A	221	P_3×P_4 RILs	Y	A
147	P_3×P_4 RILs	W	a	185	P_3×P_4 RILs	Y	A	222	P_3×P_4 RILs	Y	A
148	P_3×P_4 RILs	W	a	186	P_3×P_4 RILs	Y	A				
149	P_3×P_4 RILs	W	a	187	P_3×P_4 RILs	Y	A				
